# Screening for hepatitis C in a general adult population in a low-prevalence area: the Tromsø study

**DOI:** 10.1186/s12879-019-3832-7

**Published:** 2019-02-26

**Authors:** H. Kileng, T. Gutteberg, R. Goll, E. J. Paulssen

**Affiliations:** 1 Gastroenterology and Nutrition Research Group, Department of Clinical Medicine, UiT The Arctic University of Tromsø, 9037 Tromsø, Norway; 2 Department of Internal Medicine, Section of Gastroenterology, University Hospital of North Norway, Tromsø, Norway; 3 Research group for Host Microbe Interactions, Department of Medical Biology, UiT The Arctic University of Norway, Tromsø, Norway; 4 Department of Microbiology and Infection Control, University Hospital of North Norway, Tromsø, Norway

**Keywords:** Epidemiology, Hepatitis C, Norway, Population surveys, Prevalence

## Abstract

**Background:**

Chronic hepatitis C virus (HCV) infection can progress to cirrhosis and end-stage liver disease in a substantial proportion of patients. The infection is frequently asymptomatic, leaving many infected individuals unaware of the diagnosis until complications occur. This advocates the screening of healthy individuals. The aim of this study was to estimate the prevalence of HCV infection in the general adult population of the municipality of Tromsø, Norway, and to evaluate the efficiency of such an approach in a presumed low-prevalence area.

**Methods:**

The study was part of the seventh survey of the Tromsø Study (Tromsø 7) in 2015–2016. Sera from 20,946 individuals aged 40 years and older were analysed for antibodies to HCV (anti-HCV). A positive anti-HCV test was followed up with a new blood test for HCV RNA, and the result of any previous laboratory HCV data were recorded. Samples positive for anti-HCV and negative for HCV RNA were tested with a recombinant immunoblot assay. All HCV RNA positive individuals were offered clinical evaluation.

**Results:**

Among 20,946 participants, HCV RNA was detected in 33 (0.2%; 95% CI: 0.1–0.3), of whom 13 (39.4%; 95% CI: 22.7–56.1) were unaware of their infection. The anti-HCV test was confirmed positive in 134 individuals (0.6%; 95% CI: 0.5–0.7) with the highest prevalence in the age group 50–59 years. Current or treatment-recovered chronic HCV-infection was found in 85 individuals (0.4%; 95% CI: 0.3–0.5) and was associated with an unfavorable psychosocial profile.

**Conclusion:**

In this population-based study, the prevalence of viraemic HCV infection was 0.2%. A substantial proportion (39%) of persons with viraemic disease was not aware of their infectious status, which suggests that the current screening strategy of individuals with high risk of infection may be an inadequate approach to identify chronic HCV infection hidden in the general population.

## Background

Chronic infection with hepatitis C virus (HCV) is a leading cause of liver cirrhosis, resulting in increased risk of liver failure, hepatocellular carcinoma (HCC) and premature death [1]. Globally, an estimated 71 million people are living with viraemic HCV infection (HCV RNA positive) [2]. Norway is a low-prevalence country in this respect, as are most other Western European countries. There are uncertainties regarding the prevalence of HCV infection in Norway, as population-based data is limited. A cross-sectional study based on the Oslo Health Study in 2001 included 11,456 individuals and reported a prevalence of anti-HCV and HCV RNA of 0.7 and 0.5%, respectively [3]. In Sweden and Denmark, the estimated prevalence of chronic HCV infection is 0.36 and 0.38%, respectively [4, 5].

The incidence of HCV infection is projected to decline, but the burden of the disease is increasing [6]. According to a recent modelling approach from Norway, the HCV incidence among people who inject drugs (PWID) peaked in 2000, and has thereafter decreased. However, the occurrence of HCV-related cirrhosis and HCC in active and former PWID is expected to increase in the coming years [7]. Prevention of late complications requires treatment in the early stages of the disease, and the availability of potent direct-acting antiviral therapies (DAAs) has provided an opportunity to reverse the rising burden of HCV-related complications [8].

Surveillance of HCV is challenging for several reasons. Individuals infected with HCV are often asymptomatic until a late stage, and it is presumed that up to half of infected individuals are unaware of their status [5, 9, 10]. A recent modelling study including 28 EU countries, estimated that only 36% of those with viraemic HCV infection have been diagnosed [11].

HCV infection in Norway has by law been a notifiable disease to The Norwegian Surveillance System for Communicable Diseases (MSIS) since 1990. The surveillance system did not distinguish between resolved and chronic HCV infection prior to 2016, when it started to include only HCV RNA positive cases [12]. Yet it still does not adequately discriminate chronic HCV infection from acute infection with subsequent spontaneous clearance. Another limitation of the MSIS registration is the low notification rate. In a study of HCV treatment uptake among people who had received opioid substitution therapy (OST), only 57% of OST patients treated for HCV infection were notified to MSIS [13]. Notifications of HCV infection may reflect testing practices rather than real occurrence of the disease, thus rendering official surveillance in Norway incomplete.

In 2016, the World Health Organization (WHO) released its first global strategy on viral hepatitis aiming to eliminate HCV as a public health threat by 2030, including an 80% reduction in new HCV infections and a 65% reduction in HCV liver-related mortality, requiring diagnosis of 90% and treatment of 80% of chronically infected patients [14]. Several measures are necessary to achieve these goals. Ideally, screening for HCV infection should identify asymptomatic, infected persons before they develop cirrhosis and cirrhosis-related complications. The subsequent early treatment would improve clinical outcomes, reduce transmission risk and thus save health costs.

Screening strategies vary in different areas, based on the local epidemiology of HCV infection. In low-prevalence countries, routine screening of the entire population has not been considered cost-effective [15–18] and the approach to prevention and control of HCV infection has focused on testing persons with risk factors. Recent studies have, however, indicated that screening of the general population may be cost-effective compared to risk-based screening [19, 20]. In Norway, a limited screening of high-risk individuals is recommended, such as current or previous PWID, recipients of blood products prior to 1992, patients infected with human immunodeficiency virus (HIV), haemodialysis patients, incarcerated individuals, children born to HCV-infected mothers, individuals with elevated alanine aminotransferase (ALT), and refugees from endemic regions [21].

In the new treatment landscape with highly effective and well tolerated DAAs, many countries are reconsidering their testing strategies. Whom and how to screen has become a prioritized health policy issue.

The Norwegian Ministry of Health and Care recently launched a national strategy on viral hepatitis with aim of 90% reduction in new HCV infections by 2023 compared to 2018 [22]. Prevalence studies in the general population may be an important tool for assessing the number of infected with HCV and thus to enable an estimate of the future disease burden. The Tromsø Study is an established population survey in the municipality of Tromsø in Northern Norway, making such a prevalence study feasible.

The primary aim of the present study was to estimate the prevalence of diagnosed and undiagnosed HCV infection in the general adult population of Tromsø, Northern Norway, and second, to evaluate the efficiency of a screening approach to find individuals with undiagnosed hepatitis C infection.

## Methods

### Study population

The study was part of the seventh survey of the Tromsø Study (Tromsø 7) in 2015–2016. The Tromsø Study is a longitudinal population-based, prospective study with repeated health surveys since 1974 in the municipality of Tromsø in Northern Norway [23]. Tromsø is the largest city in Northern Norway, harbouring the world’s northernmost university, thus having a high proportion of young people. The present population (per 2nd quarter of 2018) is 76,062 inhabitants, predominantly of Norwegian origin (14% immigrants) [24].

Tromsø 7 included more than 50 research projects, covering various health issues, symptoms and chronic diseases. HCV detection was included for the first time. Based on the official population registry, residents of the municipality of Tromsø aged 40 years and older were invited to participate. A personal invitation was sent about 2 weeks before a suggested time of appointment at one permanent study site. The subjects were free to attend whenever suitable within the opening hours of the study site and within the one year duration of the study. The invitation leaflet included all necessary information, and a questionnaire was enclosed, as well as username and password for an optional online response. Non-attenders were given one reminder. Information about the survey and invitation to participate were repeatedly provided in the local newspapers.

All 32,591 citizens aged 40 years and above were invited, and 21,083 (65%) attended. Sera from 20,946 participants (64.3% of invited citizens) were tested for anti-HCV, of whom 11,004 (52.5%) were women and 9942 (47.5%) were men. The participation rate was highest in the age group 60 to 69 years for both women and men, somewhat lower in younger age groups, and lowest among those older than 80 years (Table 1).

**Table 1:** HCV testing in the Tromsø 7 Study (*n* = 20,946)

	Women		Men		Total	
Age (years)	Invited	Tested (%)	Invited	Tested (%)	Invited	Tested (%)
40–49	5195	3360 (64.7%)	5562	3033 (54.5%)	10,757	6393 (59.4%)
50–59	4534	3230 (71.2%)	4327	2767 (63.9%)	8861	5997 (67.7%)
60–69	3586	2652 (74.0%)	3543	2487 (70.2%)	7129	5139 (72.1%)
70–79	2001	1352 (67.6%)	1897	1310 (69.1%)	3898	2662 (68.3%)
80–89	981	386 (39.3%)	639	322 (50.4%)	1620	708 (43.7%)
90–104	242	24 (9.9%)	84	23 (27.4%)	326	47 (14.4%)
Total	16,539	11,004 (66.5%)	16,052	9942 (61.9%)	32,591	20,946 (64.3%)

Actual numbers for invitation to the Tromsø 7 Study, and rates (n (%)) of testing for anti-HCV according to sex and 10-year age groups

### Questionnaire

The participants responded to a self-administered questionnaire with questions about health, psychological problems triggering contact to professional health care, anxiety or depression, smoking habits, alcohol consumption, the use of drugs other than alcohol, level of education, marital status and main occupation/activity. There were two questions regarding hepatitis C (translated from Norwegian): “Have you been infected with the liver virus hepatitis C?”, and “If you have been infected with the liver virus hepatitis C: have you ever received treatment?”

### Data collection and laboratory methods

Sera from 20,946 participants were stored frozen at − 20 °C and tested for anti-HCV (ARCHITECT Anti-HCV Assay, Abbott System, Wiesbaden, Germany) at the Department of Microbiology and Infection Control, University Hospital of North Norway, Tromsø, Norway. Individuals with a positive anti-HCV test received an information letter with their test results, describing the requirement for a second blood test to discriminate between current infection and previous exposure to HCV. The second blood test was performed at the University Hospital in Tromsø, where the result was followed up by the responsible medical doctor at the Department of Gastroenterology, and compared to any existing HCV test results. Two reminders were sent to those who did not have the follow-up test. The follow-up samples were retested for anti-HCV and further tested for the presence of HCV RNA (ROCHE RT-PCR Cobas Amplicor Hepatitis C Viral Polymerase Chain Reaction, Roche Molecular System Inc., Branchburg NJ, USA). Samples positive for the anti-HCV test and negative to the HCV RNA test were analyzed with a recombinant immunoblot assay (RIBA HCV 3.0 SIA test, Chiron Cooperation, Emeryville, CA, USA) as a secondary confirmation test of the first line anti-HCV test to rule out unspecific positive tests. Samples were considered anti-HCV positive with reactivity to two or more antigens in the RIBA test, indeterminate when reactivity to only one antigen was present, which may represent previous resolved HCV-infection or unspecific antibody reactions [25], and negative when no antigen-specific reactivity was observed. The RIBA test was not carried out in cases were existing laboratory results were consistent with either spontaneous clearance (previous positive RIBA test or positive HCV RNA test followed by at least two consecutive negative HCV RNA tests with at least three months interval) or obtained sustained virologic response (SVR) after antiviral treatment. HCV genotyping was performed as a hybridization assay on products from the HCV RNA PCR according to the manufacturer’s instructions (INNO-LIPA HCV II kit, INNOGENETICS, Ghent, Belgium).

### Definitions

The term HCV exposure is used in Tables 2 and 3 to include individuals with the following characteristics: (1) persons with chronic (viraemic) HCV infection; i.e. with positive HCV RNA: (2) persons with treatment-recovered HCV infection: (3) persons with spontaneously resolved HCV infection; i.e. with positive RIBA test or positive HCV RNA test followed by at least two consecutive negative HCV RNA tests with at least three months interval: (4) persons with positive anti-HCV test, negative HCV RNA and indeterminate RIBA test.

**Table 2:** Observed and estimated prevalence of HCV exposure and chronic HCV infection

Age (years)	Invited	Tested	Observed HCV exposure (n)	Prevalence of HCV exposure (% (95% CI))	Estimated HCV exposure^*^ (n)	Observed chronic (viraemic) HCV infection (n)	Prevalence of chronic (viraemic) HCV infection (% (95% CI))	Estimated chronic (viraemic) HCV infection^a^ (n)
40–49	10,757	6393	32	0.5% (0.4–0.7)	54	5	0.08% (0.0–0.2)	8
50–59	8861	5997	69	1.2% (0.9–1.5)	102	24	0.4% (0.2–0.6)	35
60–69	7129	5139	28	0.5% (0.4–0.8)	39	4	0.08% (0.0–0.2)	6
70–79	3898	2662	3	0.1% (0.0–0.3)	4	0	0% (0.0–0.1)	0
80–89	1620	708	2	0.3% (0.1–1.0)	5	0	0% (0.0–0.5)	0
90–104	326	47	0	0% (0.0–7.6)	0	0	0% (0.0–7.6)	0
Total	32,591	20,946	134	0.6% (0.5–0.7)	209	33	0.2% (0.1–0.3)	51

Observed prevalence of HCV exposure and chronic HCV infection in the Tromsø 7 Study according to 10-year age groups. Total prevalence is corrected for different attendance rate in the different age-groups

^a^Estimated numbers of individuals in the Tromsø population is based on an equal prevalence between attenders and non-attenders. All numbers are n or proportions (%) with 95% confidence intervals (95% CI)

**Table 3:** Characteristics of the subpopulation exposed to HCV in the Tromsø 7 Study

	Tested	HCV exposed *n* = 134	HCV-antibody negative *n* = 20,812	*P* value	OR^c^ (95% CI)
Age (yrs), median (range)	20,946	54 (40–84)	56 (40–99)	*p* = 0.004	
Gender (%)	20,946			*p* = 0.199	
Male		71 (53%)	9871 (47%)		N.s.
Female		63 (47%)	10,941 (53%)		
Live with a spouse/partner (%)	19,767			*p* < 0.0005	
No		50 (43.5%)	4530 (23.1%)		N.s.
Yes		65 (56.5%)	15,122 (76.9%)		
Level of education (%)	20,573			*p* = 0.001	
< 12 years		85 (64.4%)	10,394 (50.8%)		N.s.
> 12 years		47 (35.6%)	10,047 (49.2%)		
Disability benefit recipient or unemployed (%). Retired excluded	15,870			*p* < 0.0005	2.5 (1.7–3.7)
Yes		46 (36.8%)	1973 (12.5%)		
No		79 (63.2%)	13,772 (87.5%)		
Self-reported health (%)	20,768			*p* < 0.0005	
Very bad		1 (0.8%)	73 (0.4%)		N.s.
Bad		18 (13.6%)	1065 (5.2%)		
Neither good nor bad		46 (34.8%)	5353 (25.9%)		
Good		61 (46.2%)	11,104 (53.8%)		
Excellent		6 (4.5%)	3041 (14.7%)		
Psychological problems (%)^a^	20,251			*p* < 0.0005	
Current		16 (12.9%)	879 (4.4%)		N.s.
Previous		12 (9.7%)	1801 (8.9%)		
No		96 (77.4%)	17,447 (86.7%)		
Daily smoking (%)	20,753			*p* < 0.0005	
Current		54 (40.3%)	2827 (13.7%)		4.4 (2.2–8.6)
Previous		65 (48.5%)	9129 (44.3%)		2.7 (1.4–5.1)
Never		15 (11.2%)	8663 (42.0%)		
Alcohol consumption (%)	20,816			*p* = 0.419	
4 or more times a week		5 (3.8%)	1235 (6.0%)		N.s.
2–3 times a week		30 (22.6%)	4920 (23.8%)		
2–4 times a month		48 (36.1%)	7795 (37.7%)		
Monthly or less frequently		34 (25.6%)	5067 (24.5%)		
Never		16 (12%)	1666 (8.1%)		
Use of drugs other than alcohol (%)^b^	20,498			*p* < 0.0005	
Yes, now		15 (11.5%)	65 (0.3%)		35.4 (17.4–71.9)
Yes, previously		53 (40.8%)	824 (4.0%)		15.7 (10.2–24.2)
No		62 (47.7%)	19,479 (95.6%)		

All numbers are n (%) or median (range). Chi Square, Fisher’s exact or Mann-Whitney U test were used where appropriate

^a^Psychological problems triggering contact to professional health care

^b^E.g. cannabis, amphetamines, cocaine, heroin, hallucinogens, solvents, gamma hydroxybutyrate (GHB)

^c^Results from multivariate logistic regression analysis, adjusted for age and gender, shown as odds ratios (OR) with 95% confidence intervals (CI). N.s.: Not significant

### Estimated prevalence numbers of HCV exposure and chronic HCV infection

Estimated prevalence numbers of HCV exposure and chronic (viraemic) HCV infection in the Tromsø population were calculated based on the observed prevalence in each age group and corrected for different attendance rates between the groups. An equal prevalence between attenders and non-attenders was presumed for the calculation of expected numbers of infected individuals.

### Clinical follow-up

All subjects with a positive HCV RNA test were offered a clinical evaluation, which included a thorough medical examination, the recording of the medical history and the assessment of risk factors for HCV infection. An estimate of the time-point of transmission was made based on information on the first year of high-risk behaviour, such as injecting drug use (IDU), tattoos or transfusion of blood products prior to 1992 [26]. At this stage, an additional blood sample was analysed for platelet count, alanine aminotransferase (ALT), and aspartate aminotransferase (AST) in order to calculate the Fibrosis-4 (FIB-4) index [27]. The blood sample was also analysed for hepatitis B surface antigen (HBsAg), hepatitis B core antibody (HBcAb) and antigens/antibodies to human immunodeficiency virus (HIV Ag/Ab combo). Liver stiffness (kPa) was measured with transient elastography (FibroScan® 402, Echosens, Paris, France). Significant fibrosis and cirrhosis was defined as liver stiffness values ≥7 kPa and ≥ 12,5 kPa, respectively, equivalent to METAVIR fibrosis stage ≥F2 and F4, respectively [28]. Ultrasound was performed at the responsible medical doctor’s discretion. Treatment was offered to all HCV RNA positive patients who met for clinical follow-up.

### Statistical analysis

Data summaries were performed using SPSS 24.0 software and Microsoft Excel 2013. The Chi-Square test and Mann-Whitney U test, as well as multivariate logistic regression analysis, were used to compare sociodemographic and behavioral characteristics between HCV exposed and anti-HCV negative. The Fisher’s exact test was used to test the differences between groups in case of small sample numbers. A two-tailed *p*-value < 0.05 was considered statistically significant.

## Results

### Prevalence of hepatitis C

**Fig. 1:**
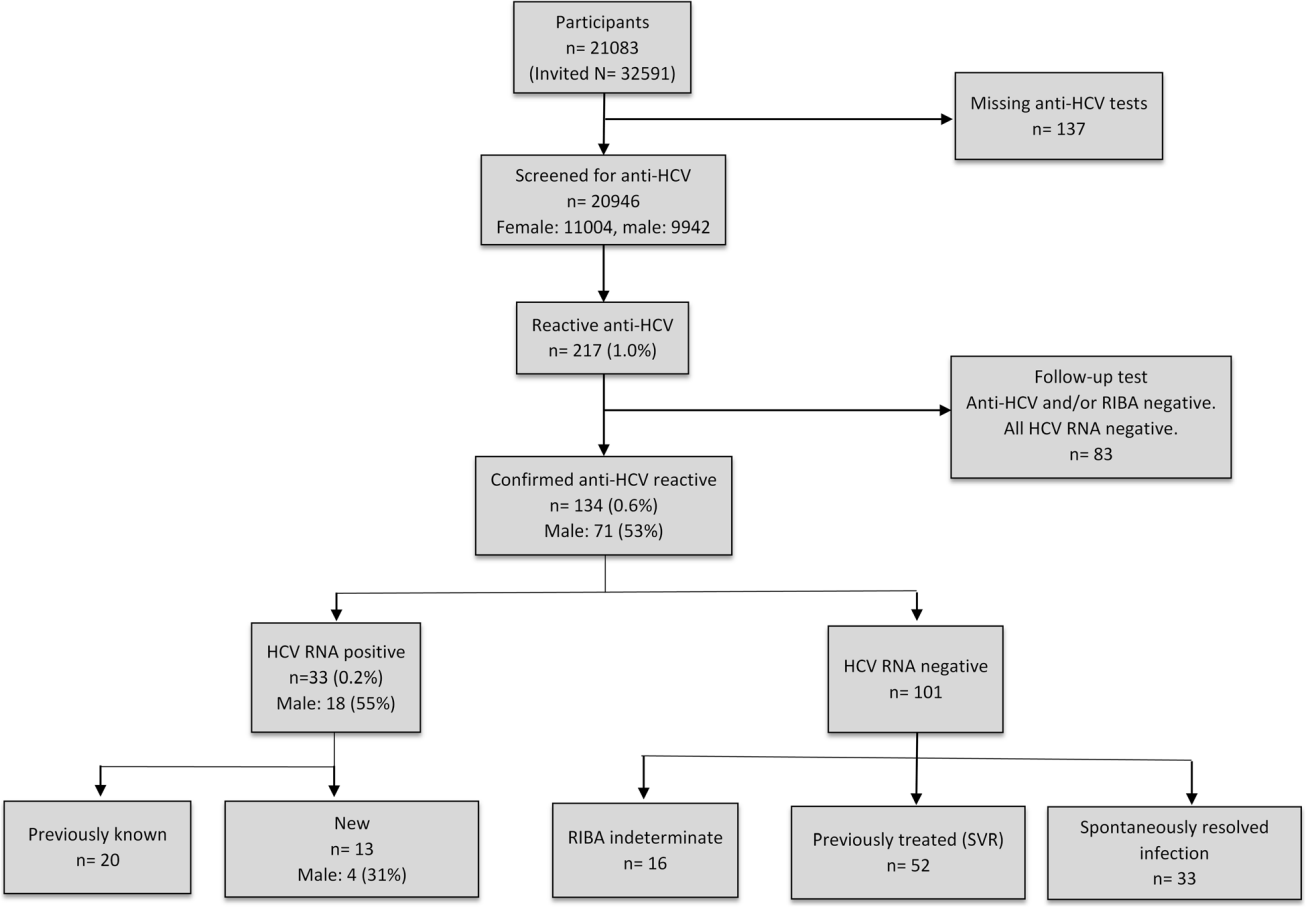
Flow chart of study design and results

Figure 1 shows a flowchart of the study with associated results. The anti-HCV screening test was positive in 217 (1.0%) of 20,946 individuals. The follow-up test was negative for anti-HCV and/or RIBA in 83 samples. Thus, the prevalence of confirmed anti-HCV was 0.6% (95% CI: 0.5–0.7%) (*n* = 134), with a sex distribution of 71 (53%) men and 63 (47%) women. HCV RNA was detected in 33 (0.2%; 95% CI: 0.1–0.3) of 20,946 participants, 18 male (54.5%). Of these viraemic cases, 13 (39.4%; 95% CI: 22.7–56.1) were not aware of their infection. Two of the 33 persons with current positive HCV RNA reported that they had received antiviral treatment earlier, one of whom had interrupted the treatment before scheduled treatment-end and was considered to be a treatment failure. The second person did not meet for clinical follow up, rendering it unclear whether the vireaemia represents reinfection or treatment failure. Overall, current or treatment-recovered HCV infection was found in 85 (0.4%; 95% CI: 0.3–0.5) of 20,946 individuals, 48 (56.5%) men and 37 (43.5%) women. Of those, 52 (61.2%) had previously received antiviral treatment with achieved SVR.

Spontaneous clearance of HCV was demonstrated in 33 (24.6, 95% CI: 17.3–31.9) of 134 anti-HCV positive individuals. The RIBA test was indeterminate in 16 of the cases that were anti- HCV positive and HCV RNA negative.

Table 2 shows the observed prevalence of HCV exposure and chronic (viraemic) HCV infection according to sex and 10-year age groups, as well as the estimated over-all prevalence. The highest prevalence of HCV exposure (1.2%) and chronic HCV infection (0.4%) was seen in the age group 50–59 years.

### HCV genotype

Data on HCV genotype (GT) was available in 75 of the 85 persons with current or recovered chronic HCV infection. HCV GT 1a was detected in 19 (25.3%) individuals, GT 1b in 10 (13.3%), GT 2b in 10 (13.3%), GT 3a in 33 (44%), GT 4 in one (1.3%) and GT 1 not available for subtyping in 2 (2.7%).

### Unawareness of HCV infection

Thirteen of the 33 (39.4%) individuals with viraemic HCV infection were not aware of their infectious status, corresponding to a population prevalence of 0.06% (95% CI: 0.03–0.09). Nine of the 13 were women and the median age was 55 years. The distribution of genotypes 1 through 3 was six GT 1a, two GT 2 and five GT 3a. The median ALT value was 48 U/L (range 21–276 U/L), with eight of thirteen persons demonstrating a normal ALT value. The median liver stiffness value was 6.7 kPa (range 4.1–17.6). Liver stiffness values were < 7 kPa in five persons and between 7 and 12 kPa in six persons. Two persons had liver stiffness > 12.5 kPa, one of whom was considered to have established liver cirrhosis based on liver stiffness of 17.6 kPa and signs of cirrhosis on ultrasound. The second person had probable liver cirrhosis based on liver stiffness of 12.6 kPa and a FIB-4 index of 3.86. HBsAg was negative in all, and HBcAb was detected in three of the 13 persons. In the self-administered questionnaire, six of the 13 individuals reported current (*n* = 2) or past (*n* = 4) drug injection. In the follow-up examination, an additional three persons reported past drug injection, thus 69.2% reported a history of IDU. The median estimated time from infection to diagnosis was 30 years (range 10–40 years). Extrapolating the observed proportion of individuals who were unaware of their HCV infection to the whole population of Northern Norway (484,001 inhabitants) implies that 290 persons above 40 years of age in this region could be unaware of ongoing HCV infection.

All 13 persons with previously undiagnosed chronic HCV infection have been successfully treated with achieved SVR 12 or 24 weeks after completed treatment.

### Factors associated with HCV exposure

Frequencies of socio-demographic characteristics in the HCV-exposed cohort compared to the background population are shown in Table 3. In univariate analysis, there was a positive association between HCV exposure and self-reported bad health, daily smoking, the use of drugs other than alcohol, lower level of education, living without a spouse/partner, being disabled or unemployed, and having psychological problems triggering contact to professional health care. In a separate question about anxiety or depression, the HCV-exposed cohort scored higher than the background population (data not shown). We found no association between alcohol consumption and HCV infection. In a multivariate logistic regression analysis including significant variables from the univariate analysis and adjusting for age and gender, significant independent predictors of being exposed to HCV were: Being disabled or unemployed (OR 2.5; 95% CI 1.7–3.7), current daily smoking (OR 4.4; 95% CI 2.2–8.6), previous daily smoking (OR 2.7; 95% CI 1.4–5.1), current use of drugs other than alcohol (OR 35,4; 95% CI 17.4–71.9), and previous use of drugs other than alcohol (OR 15.7; 95% CI 10.2–24.2).

### Estimated cost of screening

Table 4 shows the estimated costs for the screening project. The total cost for screening of 20,946 individuals was NOK 1177705 (€ 125,175), and the cost per newly detected chronic HCV infection (*n* = 13) was NOK 90593 (€ 9629).

**Table 4:** Cost of screening for HCV in the Tromsø 7 Study

	Cost per analysis	Analyses (n)	Total cost	Cost per newly detected chronic HCV infection (n = 13)
Reagents^a^		20,946	NOK 922705 (GBP 86,987, € 98,071)	NOK 70977 (GBP 6691, € 7544)
Labour costs^b^			NOK 180000 (GBP 16,969, € 19,132)	NOK 13846 (GBP 1305, € 1472)
Other^c^			NOK 75000 (GBP 7071, € 7972)	NOK 5769 (GBP 544, € 613)
Total	NOK 56.23 (GBP 5.30, € 5.98)	20,946	NOK 1177705 (GBP 111,028, € 125,175)	NOK 90593 (GBP 8541, € 9629)

Estimated costs for the HCV screening project in Norwegian Kroner (NOK), Pounds (GBP) and Euros (€). 1 GBP and 1 € approximated 10.69 and 9.48 NOK, respectively, as of 2. October 2018. 1 € = 1.15 US Dollars

^a^Reagents (anti-HCV test kits) for this study were provided by Abbvie AS, Norway. Cost is based on prices for test kits used for daily routine HCV testing

^b^Labour costs in this study were covered by the Northern Norway Regional Health Authorities with the sum mentioned, which was based on estimated time used for testing the samples in the study

^c^Participation fee for the Tromsø 7 Study

## Discussion

We have carried out a population-based screening for HCV infection in a presumed low-prevalence area. In clinical practice, the identification of individuals with viraemic HCV infection is most important. For surveillance purposes, however, reliable data for both current infection and recovered disease, either spontaneously or through treatment, is of interest. In this survey of individuals aged 40 years and older, the prevalence of chronic (viraemic) HCV infection was 0.2%. In comparison, the last population survey in Norway in 2001, including people aged 30 years and older, revealed a prevalence of chronic HCV infection of 0.5%, an estimate which also included treatment-recovered cases [3]. In our study, the prevalence of current and treatment- recovered chronic HCV infection was 0.4%, of which a high proportion (61.2%) had already received treatment with achieved SVR. A modelling study in 2013 estimated the viraemic prevalence in Norway to be 0.43% [29]. The slightly higher estimate in this study compared to ours might partly be explained by different study designs, where the modelling study was based on historical data and expert opinions.

The present study revealed that a substantial proportion (39.4%) of individuals with chronic HCV infection were unaware of their infectious status, a finding which is in line with the results of others [5, 9–11, 29]. Of the 13 previously undiagnosed individuals, 69% had a history of IDU, thus should theoretically have been detected by a risk-based screening strategy. This suggest that the current recommendation of risk-based screening is suboptimal in identifying all chronically infected persons hidden in the general population. One reason for this is that infected persons may not consider themselves as being at risk for HCV infection, i.e. persons with occasional drug use, especially in the remote past, and individuals who received blood transfusion before 1992 [9, 17]. Others have pointed out that the stigma associated with IDU; and the socio-demographic characteristics of PWIDs, create barriers that impede testing and linkage to care in this population [30].

### Strengths and weaknesses

The strength of this study is the large sample size in a general population, which enhances the probability that the study population is representative of the general population. However, there are important limitations. First, The Tromsø 7 study only included individuals aged 40 years and older. This age restriction was inherent to the overreaching study design of Tromsø 7, but clearly introduces a selection bias. IDU is the main mode of transmission of HCV [31], and it is estimated that 29.8% (range 25.0–34.8) of PWIDs in Western Europe are younger than 25 years [32]. In the municipality of Tromsø, it is estimated that the number of PWIDs is approximately 300 (personal communication, Inger Hilde Trandem, MD, Social Medical Center, Tromsø, May 28, 2018*)*. There is no clear data on their age distribution, but it is reasonable to assume that the proportion of young PWID in Tromsø is comparable to the findings in the above mentioned study. Due to the age restriction, the prevalence of HCV infection in our study is most likely underestimated.

Second, even if participation rates were generally high across all age groups, self-selection is still an important issue that may affect the representativeness of the study sample. The attenders in population surveys tend to be more educated and have a healthier life style than non-attenders [33]. The second survey of the Tromsø study (Tromsø 2) showed that various psychiatric disorders and alcohol abuse were significant predictors of nonattendance in health surveys [34], and a Canadian study demonstrated that non-response bias is a problem in alcohol and drug use surveys [35]. It is therefore reasonable to assume that current and former PWIDs are less likely to participate in health surveys, also resulting in underestimation of the true HCV prevalence and reducing the efficiency of screening in the general population.

The interpretation and significance of indeterminate RIBA reactions are unclear. In one study, 4.9% of RIBA indeterminate cases were found to be HCV RNA positive [36]. Still, most individuals with indeterminate RIBA have a negative HCV RNA test, which may represent previous resolved HCV-infection as well as unspecific antibody reactions [25]. Reports have shown that approximately half of those with indeterminate RIBA have a resolved HCV infection [37, 38]. In this study, we have chosen to include persons with RIBA indeterminate result in the HCV-exposed cohort, which could have led to overestimation of anti-HCV positive. However, the number of RIBA-indeterminate records was low, making the contribution of these less important.

### Screening strategies in a low-prevalence area: Whom and how to screen

Our study was integrated in an established population-based survey with repeated health surveys since 1974. The attendance rate was 64.3% and the estimated cost per newly detected chronic HCV infection was approximately NOK 90000 (€ 9629). HCV-screening of the general population outside such an established population survey would have been more laborious and at an expected considerably higher costs, thus making it less feasible. As discussed above, it is likely that persons belonging to risk groups for HCV infection attended the study to a lesser degree than the general population, reducing the efficiency of such an approach. On the other hand, the study has unmasked several individuals with chronic HCV infection that did not define themselves as belonging to known risk groups. A recent Spanish pilot study for an eventual population-based screening program included the adult population (20–75 years) in a small health area with a participation rate of 46.2% (2637/5706) [39]. HCV RNA was detected in 13 persons (0.5%), of whom five were unaware of the disease.

In low-prevalence countries, routine screening of the entire population has not been considered to be cost-effective [15–18], and screening are limited to high-risk populations. However, the high proportion of undiagnosed HCV infection clearly underscores the limitations of the risk-based screening approach and the need to reconsider screening strategies in order to achieve the diagnosis rate of 90% promoted by the WHO.

In the US, it is recommended a one-time screening of persons in the high-prevalence 1945–1965 birth cohort, in addition to targeted risk-based testing [40]. In the present study, the highest prevalence of anti-HCV and viraemic HCV infection was found in the age group 50–59 years, i.e. in people born between 1956 and 1965, which may be explained by a later onset of the epidemic of IDU in Norway, with a gradual increase in the number of PWID from the onset of IDU in 1973 until a peak was reached in 2000 [7]. In a birth-cohort analysis, 73% of the HCV-infected population in Norway was born between 1955 and 1980 [41]. A systematic review including several countries concluded that screening of birth cohorts, drug users, and high-risk populations was cost-effective [18]. However, recent studies indicate that universal screening of the general population may be an effective strategy. In France, where the prevalence of chronic HCV infection is 0.3% [2], a modelling study showed that universal screening of all individuals aged 18–80 years was the most effective screening strategy, and also the most cost-effective, assuming rapid initiation of treatment after diagnosis [19]. Likewise, in Spain with an HCV RNA prevalence 0.35–0.41%, a recent modelling study concluded that screening of the general adult population would identify a larger number of additional individuals with chronic HCV infection than screening high-risk groups or screening the age-cohort with the highest anti-HCV prevalence plus high-risk groups [20].

Others suggest strategies to improve targeted screening of people in high-risk groups in various settings. Primary care practitioners can play an important role in targeted screening, especially in former PWID, whereas screening of current PWID is more appropriate in settings like outpatient clinics, opioid substitution programs, jails, and psychiatric clinics [17, 42–46]. In a screening and medical follow-up programme in Northern Norway, primary care practitioners were encouraged to screen patients with former or present risk factors for HCV infection, which led to an increase in the number of newly diagnosed HCV infections in the subsequent years [47]. Technical bottlenecks in HCV testing can lead to missed opportunities in the HCV cascade of care, e.g., when a high proportion of anti-HCV positive individuals are not followed up with a confirmatory test for HCV RNA [48]. The availability of a new point-of-care (PoC) test with high sensitivity and specificity (close to 100%) for detection of HCV RNA might contribute to simplification of HCV testing and thus enable decentralisation of HCV care and treatment [49]. New technology, such as the use of dried blood spot and saliva sampling could increase access to HCV testing, e.g. in people with difficult venous access [50, 51].

There are potential negative effects associated with screening large numbers of persons in a population with low prevalence of HCV infection. In our study, the proportion of false positive anti-HCV tests was 38.2% (83/217). False-positive results can cause harm by way of anxiety and stigmatization, although such effects are difficult to quantify [52].

### Implications

Modelling studies have indicated that screening in the general adult population may be an effective screening strategy [19, 20]. Universal screening may allow diagnosis and treatment of asymptomatic infected persons, avoiding the development of complications and onward transmission, thus saving health costs. To be effective, people with the highest risk of infection must also attend the screening project. Based on this, strategies to improve targeted screening of people in high-risk groups in various settings, including primary care-based interventions, may still be the most effective approach in low-prevalence regions. To overcome the high costs associated with screening in the general population, the use of a birth-cohort screening strategy could be considered, which in our case would be based on the finding of the highest prevalence of anti-HCV and chronic HCV infection in people born between 1956 and 1965. Finally, implementation of simplified testing methods may increase access to HCV testing in both risk groups and birth cohorts.

## Conclusion

In this population-based survey the prevalence of chronic HCV infection in the general population in Tromsø was 0.2%, but due to biases the true prevalence is likely higher. A substantial proportion (39.4%) of individuals with viraemic infection was not aware of their diagnosis, suggesting that the current recommendation of screening of individuals with high risk of infection is an inadequate approach to identify all chronically infected persons. Strategies to improve HCV awareness and case-finding are needed, and for some communities, testing the general population may be a sensible approach.

## Abbreviations

ALT: alanine aminotransferase
Anti-HCV: antibodies to HCV
DAA: direct acting antiviral
GT: genotype
HCV: hepatitis C virus
HIV: human immunodeficiency virus
IDU: injecting drug use
PWID: people who inject drugs
RIBA: recombinant immunoblot assay
SVR: sustained virologic response
WHO: World Health Organization
